# Cinnamon Bud Extract Is a Source of Biomolecules Active against the Aggregation and Condensation of Alzheimer′s-Associated Tau Protein

**DOI:** 10.1021/acs.jafc.5c17659

**Published:** 2026-04-02

**Authors:** Giovanna Viola, Andrea Sperotto, Alessandro Palmioli, Roberto Tira, Francesca Munari, Michael Assfalg, Cristina Airoldi, Mariapina D’Onofrio

**Affiliations:** † Department of Biotechnology, 19051 University of Verona, Verona 37134, Italy; ‡ BioOrgNMR Lab, Department of Biotechnology and Bioscience, University of Milano-Bicocca, Milano 20126, Italy; § NeuroMI, Milan Center for Neuroscience, University of Milano-Bicocca, Milano 20126, Italy

**Keywords:** tau protein, cinnamon bud biomolecules, cinnamaldehyde, shikimic acid, protein aggregation, inhibition, NMR, liquid–liquid phase separation, tauopathies

## Abstract

Abnormal accumulation of tau fibrillar aggregates is a hallmark of tauopathies, including Alzheimer’s disease. Targeting tau aggregation represents a promising strategy for preventing and treating neurological disorders, especially using natural compounds with favorable safety profiles. In this study, we investigated a hydroalcoholic extract of *Cinnamomum cassia* buds (BCHE) and its major components, cinnamaldehyde and shikimic acid, for their effects in modulating tau repeat domain aggregation and liquid–liquid phase separation. In vitro results show that BCHE and cinnamaldehyde inhibit tau aggregate maturation, promoting the formation of nonfibrillar, off-pathway species and modulating condensate formation. These alternative aggregates exhibit reduced cytotoxicity in SH-SY5Y neuroblastoma cells and lower seeding capacity than canonical fibrils. BCHE also contains compounds capable of binding preformed tau fibrils. Overall, these findings suggest a novel mechanism by which cinnamon-derived bioactive molecules mitigate tau aggregation and reduce its cellular toxicity, highlighting their potential as neuroprotective agents.

## Graphical abstract

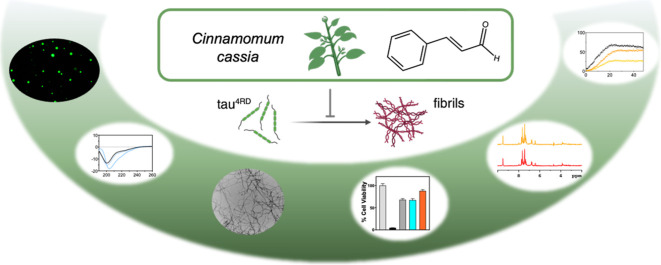

## Introduction

The abnormal deposition of protein fibrillar aggregates is a hallmark of neurodegenerative diseases (NDs). Among these, Alzheimer’s disease (AD), the most common cause of dementia, is characterized by extracellular amyloid-beta (Aβ) plaques and intracellular neurofibrillary tangles (NFTs).^1^ NFTs are composed primarily of amyloid-like filaments of hyperphosphorylated tau, a microtubule-associated protein abundantly expressed in neurons. Aberrant tau modification and accumulation are central to AD as well as other tauopathies.^2^

Initial therapeutic efforts against AD focused on Aβ have generally proven largely ineffective.^3^ Indeed, there is limited evidence that the burden of amyloid plaques meaningfully affects cognitive function or that their removal leads to positive clinical outcomes. Analyses of post-mortem tissues and associated clinical data instead suggest a correlation between the deposition of neurofibrillary tangles and cognitive impairment.^4^ As an exception, treatments with monoclonal antibodies lecanemab and donanemab have demonstrated some disease-modifying effects.^5^ However, given the limited success of Aβ-targeting therapies, attention has also shifted to the tau protein.^6^ Tau pathology correlates more closely with disease progression than Aβ, and its involvement in multiple neurodegenerative conditions underscores its therapeutic potential. Moreover, the recent hypothesis^7^ of a copathogenic interaction between Aβ and tau in AD suggests that combined targeting of both biomolecules could represent a promising strategy. Interestingly, tau has been reported to coaggregate with other neurodegeneration-associated proteins. For example, recent studies show that in Parkinson’s disease and Lewy body dementia, tau and α-synuclein coaggregate, pointing to a potential synergistic mechanism in disease onset.^8^ Altogether, this highlights the multifaceted pathogenesis of tau, involving both loss- and gain-of-function, and identifies multiple therapeutic avenues, with aggregation representing one of the targetable aspects in AD.^6^

Tau protein is structurally divided into a projection domain and an assembly domain, the latter being responsible for binding to microtubules. The protein is intrinsically disordered and exists in solution as a dynamic conformational ensemble,^9^ prone to conformational changes influenced by modifications, binding partners, and cofactors.^10,11^ Within the assembly domain, hexapeptide motifs (PHF6 and PHF6^*^) exhibit a propensity for β-sheet formation and appear crucially involved in pathological aggregation,^12^ a process that leads to the formation of NFTs. In vitro studies have demonstrated that tau aggregation proceeds via a nucleation–elongation mechanism, accelerated by polyanionic cofactors such as heparin, RNA, and unsaturated fatty acids.^13,14^ In-solution studies have often centered on the aggregation-prone repeat domain (tau^4RD^ or K18) of the microtubule-binding region, which exhibits faster aggregation kinetics than the full-length protein.^15^ Emerging evidence suggests that tau also undergoes liquid–liquid phase separation (LLPS), a phenomenon that can facilitate the nucleation of aggregates.^16–19^ Several research teams have developed small-molecule inhibitors to prevent or reverse tau aggregation and its propagation.^6^

NDs often manifest clinically after significant pathological changes at the molecular and cellular levels have occurred within the brain.^20^ Targeting protein aggregation early in the disease process, before irreversible neuronal loss, represents a critical prophylactic strategy.^21^ In this respect, standard pharmacotherapies have great limitations, especially concerning their long-term application; conversely, natural compounds have raised increasing interest as promising alternatives because of their presumed safety profile and widespread consumption.^22^ Plant-derived extracts, nutraceuticals, and dietary supplements constitute a readily available source of bioactive molecules for regular consumption.^23^ The holistic and multitarget properties often attributed to natural substances make them promising candidates for addressing the complex nature of brain disorders.^24^

Cinnamon, a widely used culinary spice, has garnered attention for its potential medicinal properties.^25^ The term “cinnamon” encompasses the spices obtained from various *Cinnamomum* tree species, including *C. verum* (Ceylon cinnamon) and *C. cassia* (Chinese cinnamon). Cinnamon has been linked to diverse health benefits such as anticancer and blood-thinning properties.^26,27^ The primary bioactive compound, cinnamaldehyde, demonstrated antimicrobial and antidiabetic effects.^28^

Emerging evidence suggests that cinnamon extracts possess bioactive compounds with neuroprotective properties.^29^ Peterson et al.^30^ showed that an aqueous extract from *C. zeylanicum* inhibited tau protein aggregation in vitro. Moreover, they found that the extract induced the disassembly of recombinant tau filaments and morphological changes of AD-brain-derived PHFs. The antiaggregant activity was attributed to a proanthocyanidin trimer molecule isolated from the extract, as well as partly to cinnamaldehyde. In a subsequent study, Frydman-Marom et al.^31^ discovered that an aqueous cinnamon bark extract reduced the formation of toxic Aβ oligomers in vitro and in vivo and corrected cognitive impairment in AD animal models. More recently, Ciaramelli et al.^32^ investigated the inhibitory effects of diverse cinnamon extracts on Aβ_42_ peptide aggregation. Among these, the *C. cassia* bud hydroalcoholic extract (hereafter BCHE) and *C. cassia* bark hydroalcoholic extract demonstrated the strongest effects against peptide aggregation and toxicity development. Subsequent molecular analysis identified flavonoids and cinnamaldehydes as the primary components responsible for the observed bioactivities.

Cinnamon buds, dried, unripe fruits of the tree, are renowned for their distinct cinnamon fragrance and flavor. *C. cassia* buds are commercially available and are commonly used as whole spices in culinary applications. Although much less investigated than cinnamon bark, the chemical composition of cinnamon buds has been thoroughly characterized.^32,33^ Cinnamon bud extract exhibits a distinctive molecular composition, including an enrichment in glycosylated flavonols among other constituents, many of which are compounds with considerable bioactive potential.

Building upon the promising results obtained with BCHE against Aβ toxicity, we sought to determine its efficacy as an inhibitor of abnormal tau aggregation. This study demonstrates that BCHE and one of its major components, cinnamaldehyde, modulate tau^4RD^ aggregation and phase separation by promoting the formation of species that show reduced toxicity in cellular models. In addition, STD-NMR experiments revealed that cinnamaldehyde can bind preformed tau fibrils. Collectively, these findings provide insights into the ability of cinnamon-derived biomolecules to modulate tau transition to toxic species.

## Materials and Methods

### Chemicals

(−)-Shikimic acid (98% purity) and *E*-cinnamaldehyde (99% purity) were purchased from Carlo Erba Reagents srl (Milan, Italy). *C. cassia* bud extracts were obtained as described previously.^32^

Stock solutions of all compounds were prepared at 5 mg/mL in mQ H_2_O for shikimic acid and 40% EtOH for BCHE, while cinnamaldehyde was ready to use.

### Recombinant Tau^4RD^ Expression and Purification

The tau^4RD^ gene (between residues Q244-E372 plus initial Met) was inserted into a pET22 vector using NdeI and *Bam*HI restriction enzymes, with a stop codon to avoid the insertion of a C-terminal histidine tag. The protein tau^4RD^ was expressed in BL21­(DE3) cells grown in LB medium at 37 °C for 5 h with 0.5 mM IPTG. The pellet was resuspended in buffer 20 mM *Tris*, pH 7.5, 50 mM NaCl, 1 mM PMSF, 1 mM MgCl_2_, DNase, and protease inhibitors and sonicated for cell lysis. Protein purification was achieved by thermal treatment of the bacterial extract (100 °C for 10 min) followed by SP-ion exchange chromatography as previously reported.^15,34,35^ Protein elution was obtained at a NaCl concentration of about 150 mM.

### Biomaterials and Extracts Used in This Work

This study utilized the repeat domain of the human tau protein, tau^4RD^, which comprises residues 244–372 of the full-length polypeptide and includes two hexapeptide motifs (275–280 and 306–311) considered central to aggregation initiation (Figure 1A).^17^

**Figure 1:**
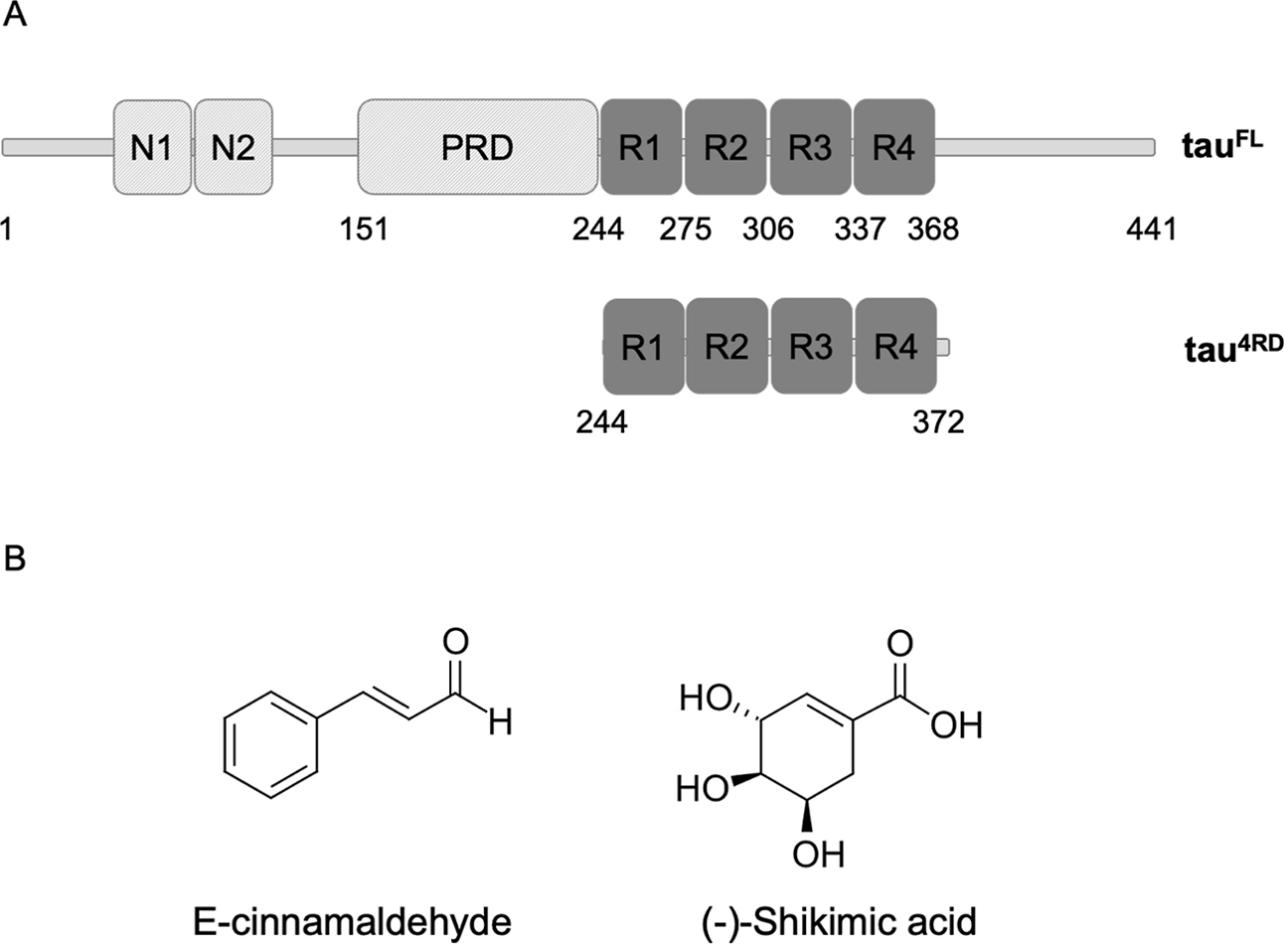
(A) Scheme of the domain structure of full-length tau, tau^FL^, and of the shorter construct, tau^4RD^, comprising the microtubule-binding repeat domains. (B) Molecular structures of cinnamon-derived molecules employed in this study.

The extract used in this work was obtained from *C. cassia* buds as previously described^32^ using a water/ethanol solution (70:30) followed by sonication. The BCHE was thoroughly analyzed by UPLC-HRMS and NMR spectroscopy by Airoldi and co-workers, and the following molecules and amounts were found, referred to 1 g of BCHE: acetate (3.5 mg), benzoic acid (0.65 mg), β-caryophyllene (12.65 mg), choline (2.31 mg), (*E*)-cinnamaldehyde (49.31 mg), cinnamic acid (11.24 mg), (*Z*)-cinnamaldehyde (0.61 mg), formate (0.52 mg), glucose (72.62 mg), glycerol (10.40 mg), 2-hydroxycinnamaldheyde (0.89 mg), methyl salicylate (17.99 mg), 2-methoxycinnamaldheyde (5.46 mg), shikimic acid (24.50 mg), succinate (0.87 mg), and sucrose (6.18 mg).^32^

### Thioflavin-T Aggregation Assay

For the thioflavin-T (ThT) assay, 10 μM*tau* ^4RD^ (filtered through a 100 kDa MWCO filter to remove any pre-existing aggregates) was diluted in 20 mM sodium phosphate buffer at pH 7.4, 50 mM NaCl, 1 mM DTT, 0.02% NaN_3_, and protease inhibitors with EDTA and incubated in the absence or presence of BCHE or different isolated molecules in 96-well dark plates at 37 °C for 48 h. Heparin and ThT were each added to the sample solutions at a 1:1 molar ratio relative to the protein. Fluorescence measurements (λ_ex_: 450 nm and λ_em_: 482 nm) were performed with a Tecan Infinite M200 Pro Microplate Reader (Tecan Group AG, Männedorf, Switzerland) with cycles of 30 s of orbital shaking at 140 rpm and 10 min of rest before the fluorescence reading during the incubation, as described in previous works.^14,36^ ThT curves were obtained on four replicates for each sample. Each replicate was fitted individually using GraphPad Prism 10 software (GraphPad Software, San Diego, California, https://www.graphpad.com/) using the following equation:

$$Y=y_{i}+m_{i}t\frac{y_{f}+m_{f}t}{1+e^{-\left[\left(t-t_{0.5}\right)/\tau \right]}}$$

where *Y* is the fluorescence intensity as a function of time *t*, *y*_i_ and *y*_f_ are the intercepts of the initial and final baselines with the *y*-axis, respectively, *m*_i_ and *m*_f_ are the slopes of the initial and final baselines, respectively, *t*_0.5_ is the time needed to reach halfway through the elongation phase, and τ is the elongation time constant.^37^ The fitted parameters from the four replicates were averaged and are reported in Table 1 as the mean ± SD. The aggregation data are shown in Figure 2 as the mean ± SD of the replicates at each time point. Differences in aggregation kinetic parameters between samples incubated with bioactive molecules and the control condition (protein alone) were analyzed using one-way ANOVA followed by Dunnett’s post hoc test, performed with GraphPad Prism.

**Table 1:** Aggregation Kinetics Parameters^a^ ^,^ ^b^

	protein only	BCHE 5 μg/mL	BCHE 10 μg/mL	shikimic acid 5 μg/mL
*t*_0.5_ (h)	9.3 ± 1.0	13.3 ± 0.5^c^	13.0 ± 1.3^c^	8.1 ± 0.1
τ (h)	4.3 ± 0.9	4.2 ± 0.3	4.6 ± 3.3	3.4 ± 0.2

a Data were obtained from ThT fluorescence assays performed on tau^4RD^ in the absence or presence of cinnamon-derived compounds, with values reported as the mean ± SD of four replicates, each fitted individually.

b *t*_0.5_: midpoint of the transition; τ: elongation time constant.

c This value is significantly different (*p* < 0.05) from the protein-only control based on one-way ANOVA analysis followed by Dunnett’s test.

**Figure 2:**
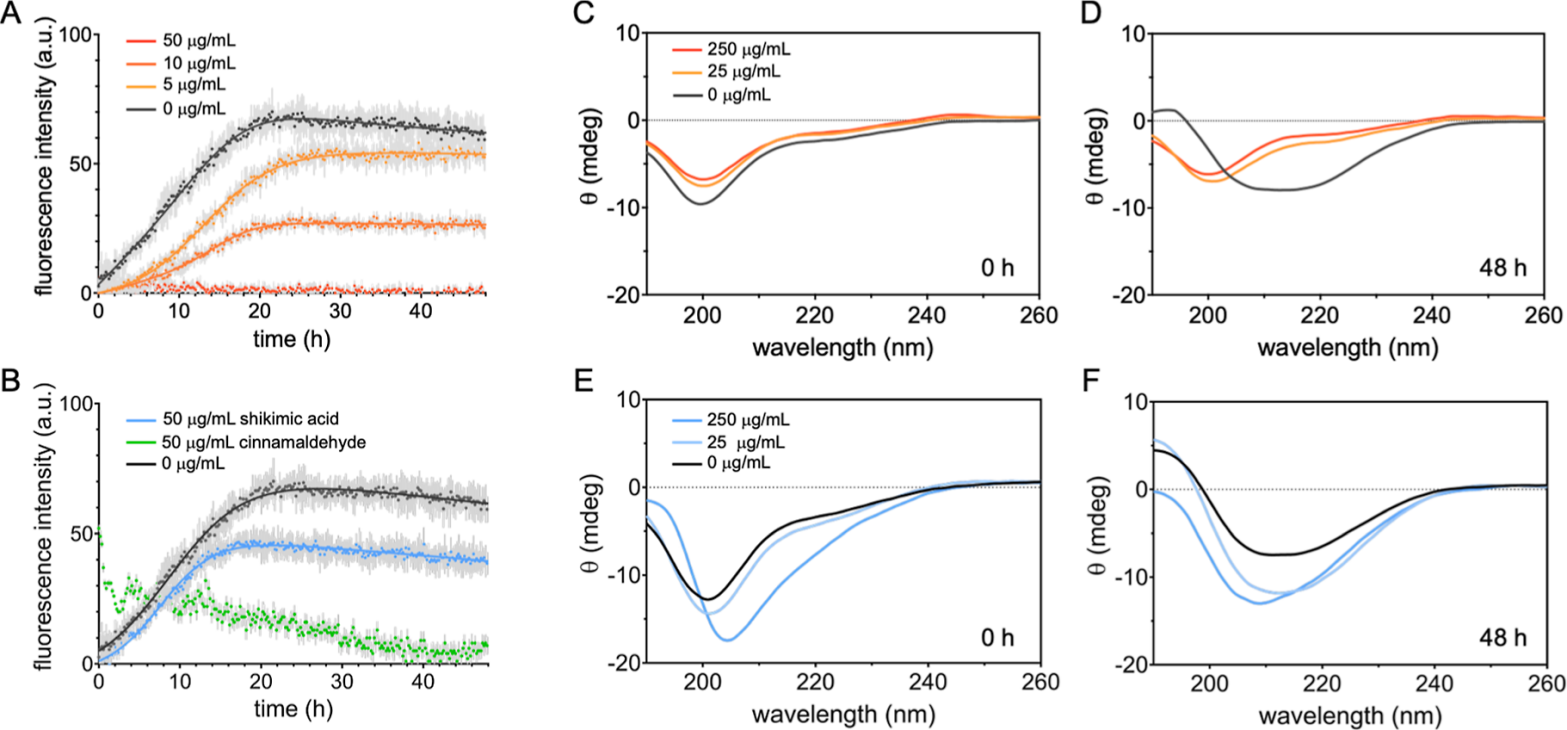
Modulation of aggregation kinetics and conformational transitions. (A,B) Aggregation kinetics monitored through thioflavin-T fluorescence; tau^4RD^ (10 μM) was incubated under aggregating conditions in the absence (black) or presence (colored data sets) of cinnamon-derived compounds; (A) samples contained BCHE at the indicated concentrations; (B) samples contained 50 μg/mL shikimic acid (28.7 μM, ∼3-fold molar excess) (blue) or cinnamaldehyde (38 μM, ∼4-fold molar excess) (green); measurements were performed in quadruplicate, data represent the mean ± SD, and solid lines correspond to the best-fit curves using an empirical sigmoidal function. (C–F) Secondary structure changes monitored by circular dichroism spectroscopy on tau^4RD^ samples (50 μM) incubated under aggregating conditions in the absence (black curves) or presence of 25 (light orange) or 250 (orange) μg/mL of BCHE, acquired before (C) and after 48 h (D) of incubation, and in the absence (black curves) or presence of 25 (0.14 mM) (light blue) or 250 (1.4 mM) (blue) μg/mL shikimic acid, acquired before (E) and after 48 h (F) of incubation.

### Sample Preparation for CD, TEM, and NMR Analysis

Solutions of 50 μM filtered tau^4RD^ protein in 20 mM sodium phosphate buffer at pH 7.4, 0.5 mM DTT, 0.02% NaN_3_, and protease inhibitors with EDTA were incubated in the absence or presence of BCHE or different isolated molecules in static conditions at 37 °C for 48/72 h using heparin at a 1:1 molar ratio as an aggregation enhancer.^14,36^

For both CD and TEM measurements, the whole sample was used before or after aggregation and diluted as specified.

For saturation transfer difference (STD)-NMR experiments, a solution of 200 μM filtered tau^4RD^ protein in 20 mM sodium phosphate buffer at pH 7.4, 0.5 mM DTT, and 0.02% NaN_3_ was incubated in static conditions at 37 °C for 48 h using heparin at a 1:1 molar ratio as an aggregation enhancer. The fibrils were separated as a pellet after centrifugation at 20,000*g* for 30 min and resuspended in 20 mM sodium phosphate buffer at pH 7.4, 0.5 mM DTT, and 0.02% NaN_3_ for NMR spectra acquisition.

### CD Spectroscopy

Solutions containing tau^4RD^ aggregates were diluted in 20 mM sodium phosphate buffer, pH 7.4, to a final concentration of 6 μM. Far-UV spectra (190–260 nm) were recorded using a Jasco J-1500 spectropolarimeter equipped with a Peltier-type cell holder for temperature control (Jasco, Easton, MD, USA) at 25 °C with a scan rate of 50 nm min^–1^, a bandwidth of 1 nm, and an integration time of 2 s in 0.1 cm cuvettes. Three spectra accumulations were collected and averaged for each sample at different times (0 and 48 h). The spectrum of the buffer alone (with or without the compounds) was subtracted from the spectrum of the corresponding sample. Data were analyzed with Spectra Manager, and graphs were generated with GraphPad Prism 10 software (GraphPad Software Inc., La Jolla, CA, USA).

### Transmission Electron Microscopy

TEM measurements were performed using 100 μL of tau^4RD^ aggregates (obtained after 72 h of incubation with or without compounds), washed, and diluted in mQ H_2_O to a final concentration of 5 μM (monomer concentration). A 30 μL portion of diluted aggregates was adsorbed onto a 400-mesh holey film grid; after staining with 2% uranyl acetate (for 2 min), the samples were observed with a Tecnai G2 (FEI) transmission electron microscope operating at 100 kV. Images were captured with a Veleta (Olympus Soft Imaging System, Münster, Germany) digital camera using FEI TIA acquisition software (Version 4.0). Fibril characteristics were analyzed using ImageJ software (version 2.0).

### NMR Spectroscopy

NMR experiments were acquired at 600 MHz on a Bruker Avance III spectrometer equipped with a triple resonance TCI cryoprobe or on a Bruker Avance NEO spectrometer equipped with a cryoprobe Prodigy TCI. All NMR spectra were processed and analyzed using Topspin 4.1.1 software (Bruker, Karlsruhe, Germany). A total of 8 transients were acquired over a spectral width of 9615 Hz and 32,768 complex points with a recycle delay of 4 s. STD experiments were acquired with 8 scans at 25 °C. Selective saturation of the protein fibrils at a 0.4 ppm frequency was carried out with a 2 s pulse train (60 Gaussian-shaped pulses of 50 ms separated by 1 ms interval) included in the relaxation delay, and a 25 ms spin-lock was used to reduce the broad background protein signal. The STD spectrum was obtained by subtracting the on-resonance spectrum (saturation at 0.4 ppm) from the off-resonance spectrum (saturation at −40 ppm). In all experiments, water suppression was obtained using the excitation sculpting pulse scheme. STD blank experiments with samples containing only BCHE were acquired as a reference to verify the binding.

### Tau^4RD^ Condensates in the Presence of BCHE, Cinnamaldehyde, and Shikimic Acid

Liquid–liquid phase separation (LLPS) of tau^4RD^ was induced using either heparin or polyuridylic acid (polyU) anions in the absence or presence of BCHE, *E*-cinnamaldehyde, and shikimic acid at different concentrations (25–250 μg/mL). For LLPS of tau^4RD^ with heparin, all samples contained 50 μM protein in 20 mM sodium phosphate buffer, pH 6.0, 30 mM NaCl, 1 mM DTT, and 11 μM heparin. After 5 min of incubation, 10 μM ThT was added to monitor fibrilization, and the samples were split to add the compounds. LLPS of tau^4RD^ with polyU was obtained by mixing 50 μM protein with 125 μg/mL polyU RNA, 1 mM DTT in 25 mM Hepes, pH 7.4, and 0.6 μM of Alexa488-tau^4RD^ as a reporter.^19^ For phase separation imaging, 7 μL of solution was spotted onto a microscope slide, covered with a circular coverslip, and sealed with nail polish. Condensate images were acquired using a Leica TCS SP5 AOBS microscope to visualize droplet formation over time in both bright-field and green channels. Image analysis was performed using FIJI ImageJ software (version 2.0).

### Sample Preparation for Cellular Viability and Seeding-Based Aggregation Assays

Tau^4RD^ aggregation reactions were performed by adding purified tau^4RD^ protein at a final concentration of 100 μM to an appropriate 0.20 μm-filtered buffer (20 mM sodium phosphate buffer at pH 7.4, 50 mM NaCl, 1 mM DTT, and protease inhibitor with EDTA), followed by incubation in the absence or presence of BCHE (500 μg/mL), cinnamaldehyde (25 μg/mL), or shikimic acid (50 μg/mL) in static conditions at 37 °C for 24 h. Protein and heparin were in a 4:1 molar ratio. After incubation, fibrils/oligomers were separated as a pellet by centrifugation at 20,000*g* for 40 min. After two washes with sterile water, the pellets were resuspended in a volume of H_2_O corresponding to that used for the aggregation mixture. All samples were verified by SDS-PAGE.

### Seeding-Based Aggregation and Immunoblot Analysis

HEK293T cells were cultured in DMEM High Glucose with stable glutamine and sodium pyruvate (Aurogene), supplemented with 10% FBS (fetal bovine serum) (Aurogene) and 2% penicillin/streptomycin solution (100×) (Aurogene) at 37 °C and 5% CO_2_ in a humidified incubator. Once the exponential growth phase was reached, HEK293T cells were counted using a Countess Automatic Cell Counter (Thermo Fisher Scientific), and 30,000 cells/well were seeded in a flat-bottomed 24-well plate. The following day, 0.5 μg/well of the pEGFP-N1 vector carrying the gene for the expression of human full-length tau P301L was transfected into HEK293T cells using PEI (polyethylenimine) (Merck). After 24 h, HEK293T cells were treated with 5 μM*tau* ^4RD^ aggregates obtained either in the absence or presence of BCHE, cinnamaldehyde, or shikimic acid, while untreated cells served as a negative control. Lipofectamine LTX at 0.5% was employed as a transfection agent. After 48 h of treatment, cells were first scraped into Triton lysis buffer (1% Triton X-100 in 50 mM *Tris*, 250 mM NaCl, pH 7.6, containing 50 mM NaF and 1 mM EDTA) supplemented with protease and phosphatase inhibitors and then incubated on ice for 30 min. Lysates were centrifuged at 20,000 g for 40 min at 4 °C. Supernatants were kept as the “Triton fraction”, whereas the pellets, corresponding to insoluble protein aggregates, were washed once in Triton lysis buffer, separated again with centrifugation, resuspended in SDS lysis buffer (2% SDS in 50 mM *Tris*, pH 7.6, 250 mM NaCl) at a volume that is 1/3 of the Triton lysis buffer, and heated for 15 min at 80 °C. After centrifugation at 20,000*g*, the supernatants were collected as the “SDS fraction”. Total soluble proteins contained in the Triton fraction were quantified with the BCA assay, and an equal volume of both the Triton fraction and the SDS fraction was loaded on SDS-PAGE. Subsequent immunoblot analysis was performed with a tau-5 antibody, specific for human tau^FL^. β-actin immunoblotting was used to verify equal protein loading in the Triton-soluble fraction. Immuno-reactive proteins were detected using the ECL Prime Western Blotting Detection Reagents (Cytiva) according to the manufacturer’s instructions.

### Cell Viability Assay

SH-SY5Y neuroblastoma cells were maintained in the same culture conditions described for HEK293T cells. Once 70–80% confluence was reached, the SH-SY5Y cells were washed with phosphate-buffered saline (PBS) buffer, collected using trypsin, and counted. 50,000 SH-SY5Y cells/well were then seeded in a flat-bottomed 96-well plate and incubated at 37 °C and 5% CO_2_ in a humidified incubator. After 24 h, cells were treated with 5 μM*tau* ^4RD^ samples obtained in the absence or presence of BCHE, cinnamaldehyde, or shikimic acid. Untreated cells were used as a negative control, while cells treated with 0.5% NaN_3_ served as a positive control. After 48 h of treatment, cells were incubated with 0.5 mg/mL of the tetrazolium salt MTT (3-(4,5-dimethylthiazol-2-yl)-2,5-diphenyltetrazolium bromide) for 3 h at 37 °C. Following MTT reduction by metabolically active cells, the resulting insoluble formazan crystals were dissolved in 200 μL of DMSO. The reduced MTT was evaluated by measuring the absorbance at 565 nm, with background correction at 640 nm. Experiments were performed in triplicate on a Tecan Infinite M200 Pro microplate reader. A one-way ANOVA analysis was performed to find significant differences. The significance threshold was set at *p*-value = 0.05.

### Statistical Analysis

Statistical analysis was applied to ThT-derived kinetic parameters, TEM-derived fibril morphology data, and cell viability measurements. The statistically significant differences between samples were determined using one-way ANOVA followed by Dunnett′s multiple comparison test, comparing the means of each sample group with those of the control group. The significance threshold was set at *p*-value = 0.05. For TEM analysis, 10–25 measurements (from different images) for each parameter were analyzed. For the cell viability assay, measurements were performed in triplicate. All sets of samples comply with the normal distribution as determined by the D′Agostino and Pearson test. Standard deviation was homogeneous according to Brown–Forsythe’s and Bartlett’s tests. For both analyses, *p* values were indicated as follows: *0.01–0.05, **0.001–0.01, ***0.0001–0.001, and **** < 0.0001.

## Results and Discussion

### BCHE and Cinnamaldehyde Impair Tau Fibril Formation via the Deposition Pathway

Tau protein aggregation is a complex process that can be modulated by many factors, including post-translational modifications, interactions with other proteins or nucleic acids, and both endogenous and exogenous compounds.^14,38^ Here, protein aggregation kinetics experiments monitored by fluorescence spectroscopy were used to investigate the influence of BCHE and cinnamon single compounds on tau fibrillar aggregate formation. Tau aggregation, commonly stimulated by cofactors like heparin, is conveniently monitored using thioflavin-T (ThT) fluorescence-based assays, which rely on ThT fluorescence enhancement after its binding to β-sheet structures. The resulting aggregation curve, generated from fluorescence measurements over time, provides information on the temporal evolution of tau aggregation. This curve typically reveals distinct phases: an initial lag phase, during which oligomeric nuclei form; an elongation phase, characterized by rapid fibril growth; and a final plateau phase, signifying the attainment of a steady-state equilibrium.

In our experiments, tau^4RD^ underwent rapid nucleation, followed by a moderately fast elongation phase with a 4 h time constant, and the transition midpoint was reached after 9 h (Figure 2A, Table 1). The addition of BCHE at 5 and 10 μg/mL increased the midpoint transition time to approximately 13 h, whereas the elongation time was not significantly affected. At a higher concentration (50 μg/mL), the ThT fluorescence intensity did not increase, suggesting that fibril formation was strongly inhibited or absent. Overall, these results indicate that BCHE delays the aggregation process and, at sufficiently high concentrations, effectively suppresses fibril formation.

A complementary examination of secondary structure changes was conducted using circular dichroism (CD) spectroscopy. The CD spectrum of tau^4RD^ exhibits a strong negative signal with a peak near 200 nm and lacks prominent bands at longer wavelengths (Figure 2C), consistent with the absence of defined secondary structure elements. The BCHE did not alter the overall spectrum profile, except for a slight reduction in peak intensity, indicating that tau^4RD^ retained its fully unstructured character under these conditions. After 48 h of incubation in the presence of heparin, the spectrum of tau^4RD^ without extract showed a clear shift to predominantly β-strand structures (Figure 2D), consistent with the formation of fibrils as detected by the ThT assay. By contrast, no conformational transition was detected for samples containing 25 or 250 μg/mL BCHE, suggesting that concentrated extract obstructs fibril formation, as observed in the ThT assay at 50 μg/mL.

A reduced maximum ThT intensity at higher extract concentrations could reflect fewer fibrils or interference with the ThT binding. To gain more insight into the types of aggregates formed, we examined the protein deposits by transmission electron microscopy (TEM). Filamentous aggregates several micrometers long were obtained in the absence or presence of low-concentration extract (Figure 3A,B). By contrast, only sparse filamentous fragments were observed on the TEM grid in the case of a high-concentration extract. The latter also presented some differences in morphology, particularly in terms of filament large width and crossover distances relative to the aggregates obtained under the other conditions (Figure 3E).

**Figure 3:**
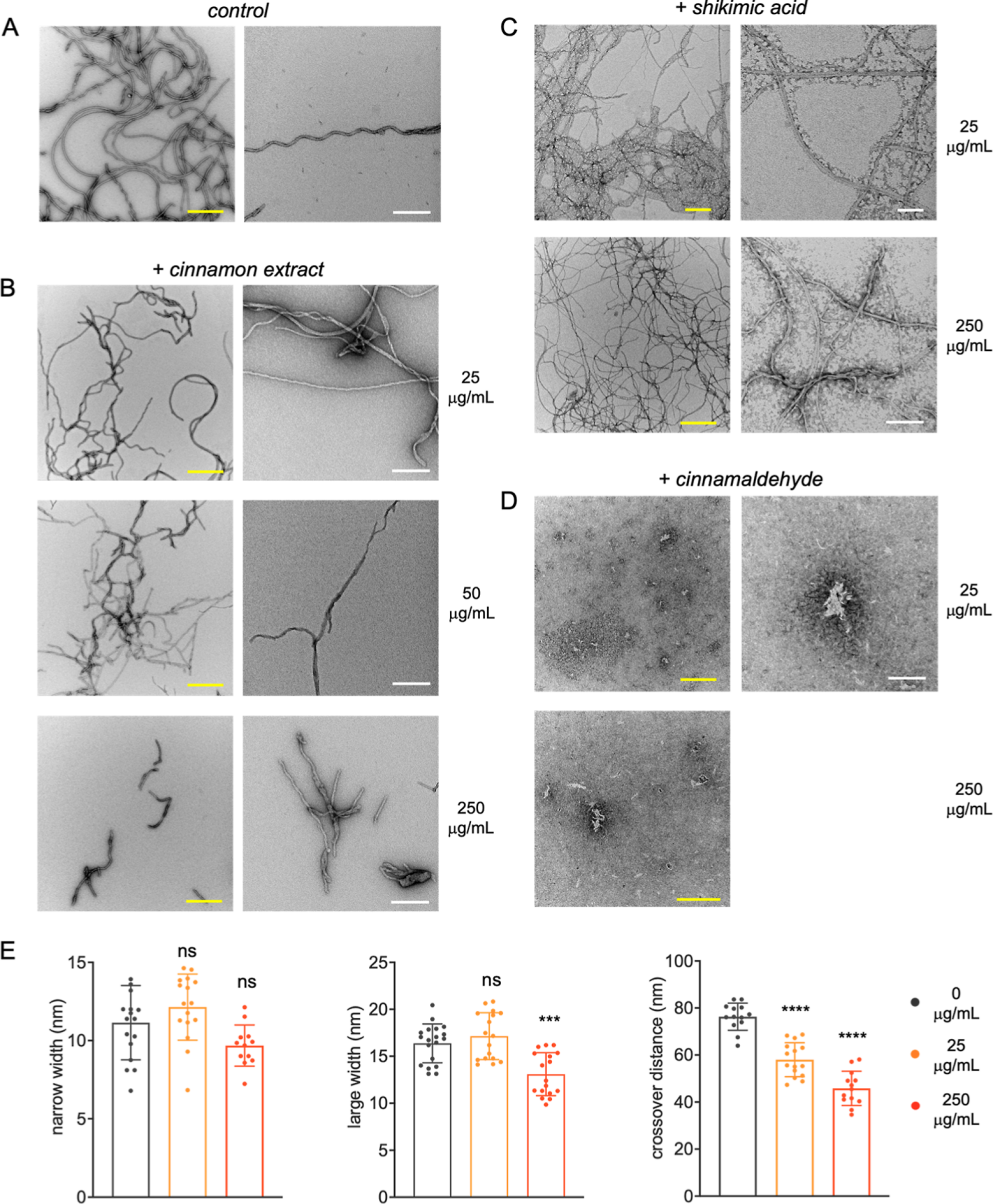
Protein aggregates morphology. Representative transmission electron microscopy (TEM) images of end-point aggregates (72 h incubation) acquired from 50 μM*tau* ^4RD^ samples containing (A) no cinnamon compounds, (B) 25, 50, or 250 μg/mL BCHE, (C) 25 or 250 μg/mL shikimic acid, and (D) 25 or 250 μg/mL cinnamaldehyde. Scale bars are 500 nm (yellow) or 200 nm (white). (E) Morphological analysis of filamentous deposits visualized by TEM for samples containing the indicated concentrations of BCHE. Statistical analysis by one-way ANOVA-Dunnett′s test: ns = nonsignificant; *p* = *** 0.0001–0.001 and **** 0.00001–0.0001.

Previous NMR analysis of the metabolic profile from BCHE revealed that, in addition to glucose, (E)-cinnamaldehyde and shikimic acid (Figure 1B) are the predominant constituents (49.31 mg and 24.50 mg, respectively, per g of BCHE).^32^ Consequently, the influence of these compounds on tau aggregation was also evaluated. However, the interference of cinnamaldehyde with ThT fluorescence (Figure 2B) and CD measurements limited the analysis for this compound to TEM observations, despite cinnamaldehyde alone showing only flat lines in both assays (data not shown). The presence of shikimic acid did not significantly alter the overall aggregation process compared to the control (Figure 2B, Table 1) and did not impair the formation of β-strand structures, as observed by CD (Figure 2E,F). Tau^4RD^ formed long, filamentous aggregates at both low and high concentrations of shikimic acid (Figure 3C). By contrast, no fibrillar-like deposits were observed when cinnamaldehyde was added to the protein solution (Figure 3D). This result is in agreement with previously reported data showing that cinnamaldehyde significantly inhibits the aggregation of a longer tau construct (spanning residues 255–441).^39^ Thus, both the concentrated BCHE and the isolated cinnamaldehyde hamper the formation of mature filamentous aggregates.

### Cinnamaldehyde Present in the Extract Shows Affinity for Tau Fibrils

The discovery of molecules interacting with preformed fibrils holds significant promise for novel therapeutics and diagnostic applications in neurodegenerative diseases.^40^ Indeed, amyloid-binding molecules can track the formation and distribution of fibrillar aggregates or they can be exploited to shift the equilibrium away from harmful oligomers by redirecting the progression of aggregation. Given the rich molecular diversity of food extracts, screening for amyloid-binding molecules could lead to the discovery of novel molecular ligands.

STD-NMR spectroscopy is a powerful technique for identifying and characterizing the binding of small molecules to biological macromolecules, even within complex mixtures.^41–43^ This capability makes STD-NMR valuable for screening compound libraries and analyzing natural product extracts, eliminating the need for extensive fractionation.^44,45^ We therefore employed STD-NMR methodology to investigate the binding of BCHE components to preformed tau^4RD^ fibrils. STD-NMR involves comparing two ^1^H NMR spectra: an “on-resonance” spectrum with protein signal saturation (avoiding ligand saturation) and an “off-resonance” spectrum with saturation far from both protein and ligand signals. Subtracting the on-resonance spectrum from the off-resonance spectrum reveals ligand signals affected by protein saturation, indicating specific small molecule-to-fibril interactions.

To investigate the interaction of BCHE with preformed tau^4RD^ fibrils, STD-NMR spectra were obtained using saturation times from 0.5 to 2 s. Irradiation at protein frequencies resulted in clear saturation of cinnamaldehyde signals (Figure 4A). The integrated intensity of the fully resolved aldehydic hydrogen signal (Figure 4B) of cinnamaldehyde was then plotted against the corresponding saturation times, generating a buildup curve (Figure 4C). This buildup curve provides insights into the efficiency of saturation transfer, which is related to the binding affinity and exchange kinetics of cinnamaldehyde with the tau^4RD^ fibrils. In the absence of tau fibrils, the control experiment showed minimal STD effects. These findings collectively demonstrate that cinnamaldehyde, within the complex milieu of the BCHE, exhibits a moderate affinity for preformed tau^4RD^ fibrils. Consequently, the cinnamaldehyde structure offers a potential lead for the design of high-affinity drug molecules. These results are consistent with our previous NMR findings, which showed that among the molecules of espresso coffee extract, both caffeine and chlorogenic acid can bind to the preformed tau^4RD^ fibrils.^46^ Notably, chlorogenic acid is a cinnamate ester, suggesting that this structural motif may play a key role in mediating the interaction with tau fibrils. A recent study reported the structure of the bioactive molecule epigallocatechin gallate (EGCG), abundant in green tea, bound to tau fibrils from AD patients.^47^ Although that cryo-EM structure does not provide atomic-level resolution, it offers valuable structural insights into the determinants of the interaction. Specifically, hydrogen bonding to tau side chains and π–π stacking interactions between the aromatic rings of EGCG molecules may represent a common mechanism underlying the recognition of tau fibrils by the biomolecules we identified in different food matrices.

**Figure 4:**
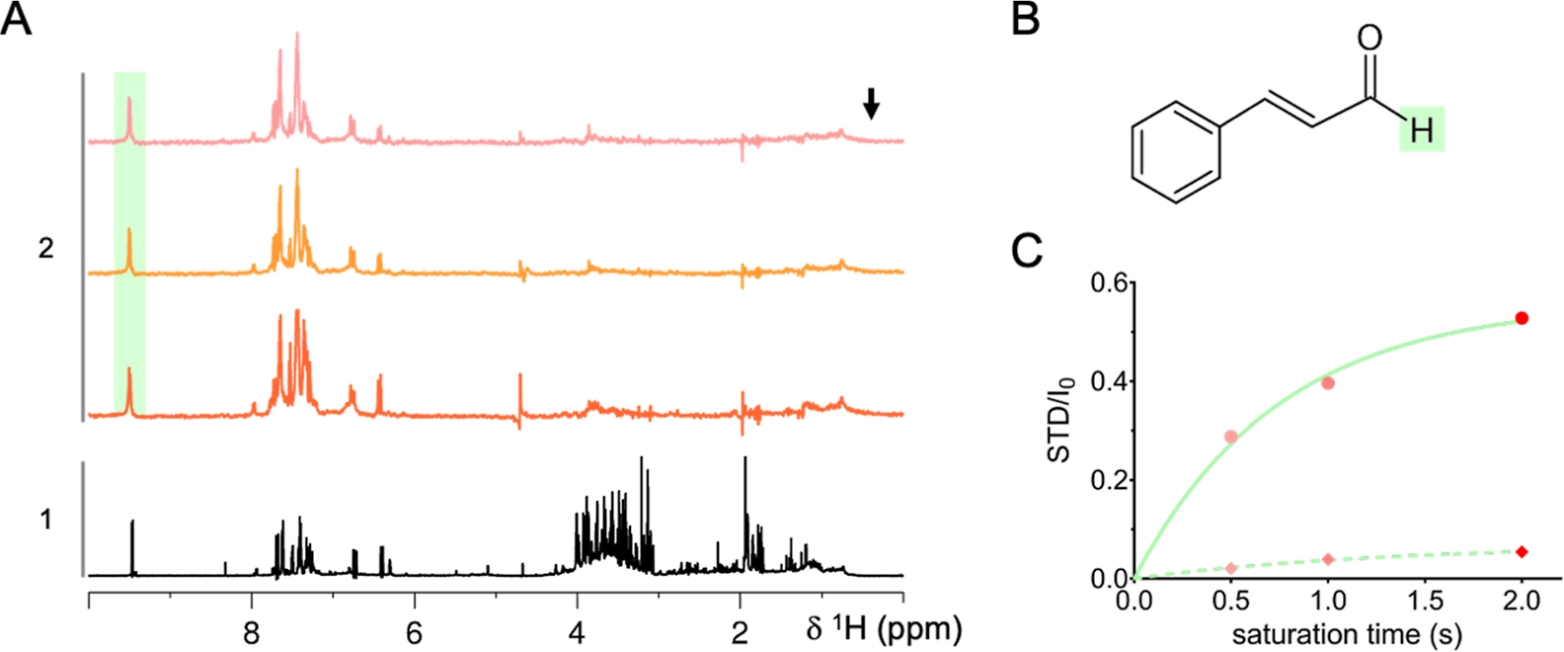
Interaction between BCHE and tau^4RD^ fibrils probed by STD-NMR. (A) ^1^H NMR spectrum of a solution containing BCHE (5 mg/mL) (1); STD-NMR spectra of a mixture of BCHE (5 mg/mL) and tau^4RD^ fibrils (80 μM) (2). The STD spectra were acquired with saturation at 0.4 ppm (indicated by the black arrow) applied for 2 s (orange), 1 s (light orange), or 0.5 s (pink). The green shaded area indicates the signal attributed to the aldehydic hydrogen of cinnamaldehyde, highlighted in panel (B). The spectra are shown as the difference of the STD spectra acquired on BCHE in the presence and absence of tau^4RD^ fibrils with the same saturation time. (C) STD intensity buildup as a function of applied saturation time. STD is the difference in intensity of the signal between the off-resonance and on-resonance spectra, and *I*_0_ is the intensity of the reference. Data were obtained in the presence (filled circles) or absence (diamonds) of tau^4RD^ fibrils. The continuous and dotted green lines represent monoexponential fits to the data. The samples were dissolved in 20 mM sodium phosphate buffer at pH 7.4, 0.5 mM DTT, and 0.02% NaN_3_. The spectra were acquired at 25 °C.

### Impact of BCHE, Cinnamaldehyde, and Shikimic Acid on Tau-Induced Cytotoxicity and Aggregation

The collected data indicate that BCHE and cinnamaldehyde can impair tau fibril maturation, favoring the formation of short filaments or amorphous aggregates, while shikimic acid did not impair the formation of β-strand structures.

Previous studies have reported that tau oligomers reduce cell viability and represent the cytotoxic species that are critical for disease progression.^48,49^ Therefore, we aimed to investigate whether the nonfibrillar species formed after treatment of tau with food-derived molecules exert a cytotoxic effect. To this end, we treated the human neuroblastoma cell line SH-SY5Y with tau^4RD^, aggregated in the presence of BCHE, cinnamaldehyde, or shikimic acid (Figure 5A). The toxicity was evaluated using the MTT-based colorimetric assay, which measures the formation of the insoluble purple formazan by live cells. Cell viability decreased to ∼75% after treatment with tau^4RD^ fibrils (Figure 5B), consistent with previously reported data on the cytotoxicity of tau aggregates.^50^ A similar effect on SH-SY5Y cells was observed following treatment with aggregates formed in the presence of shikimic acid (Figure 5B), in line with its inability to inhibit fibril formation. By contrast, treatment with BCHE significantly reduced the toxicity of tau^4RD^ aggregates, increasing cell viability at ∼90%, whereas treatment with cinnamaldehyde completely restored cell viability (Figure 5B).

**Figure 5:**
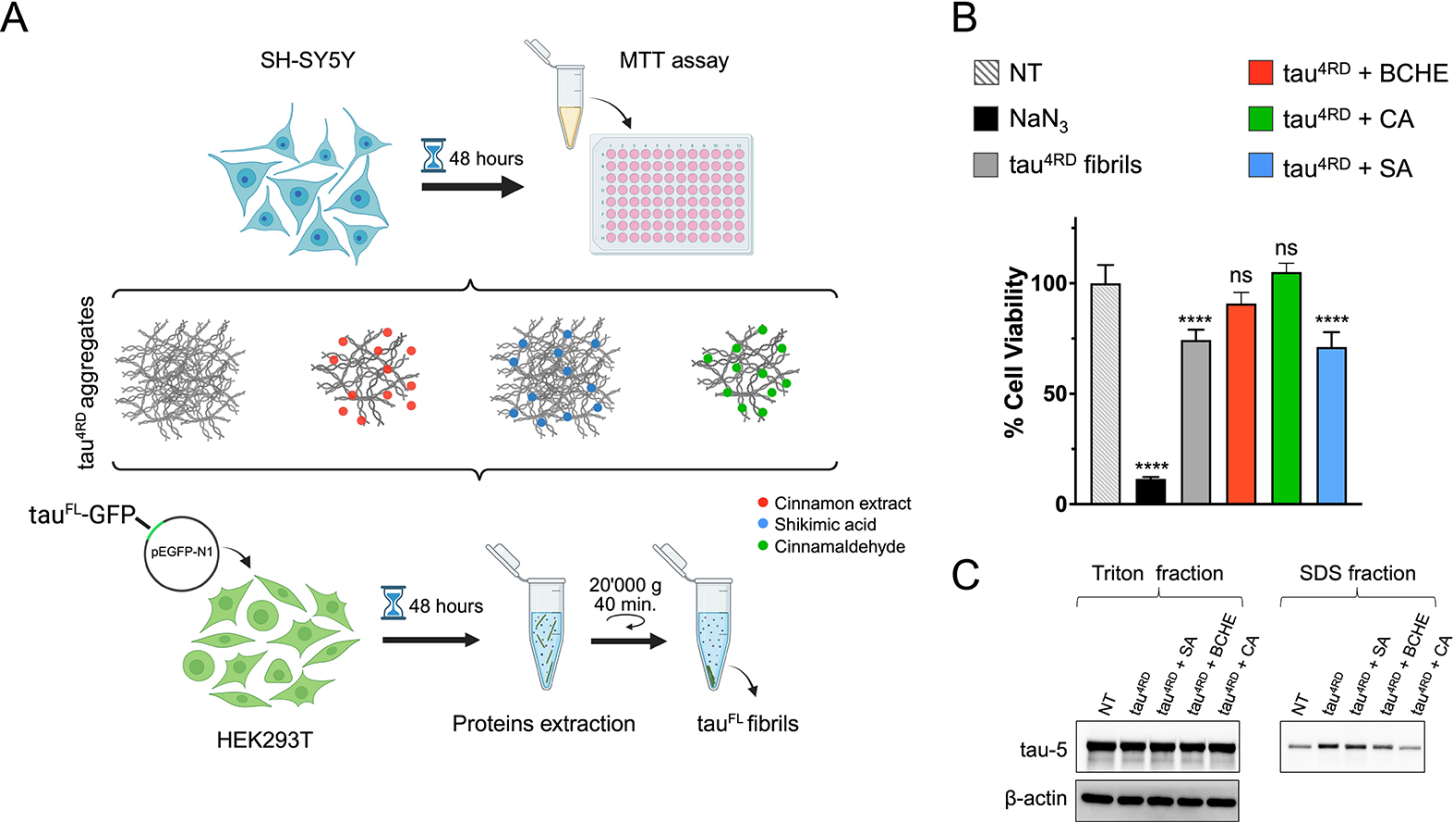
(A) Schematic depiction of experimental design of cell viability and seeding-based aggregation assays in cellular models. (B) Cell viability assay performed on SH-SY5Y neuroblastoma cells not treated (NT) or treated for 48 h with tau^4RD^ fibrils (aggregates) obtained in the absence or in the presence of BCHE, cinnamaldehyde (CA), or shikimic acid (SA). One-way statistical analysis ANOVA, followed by Dunnett’s multiple comparison test, was performed to compare each treated sample with the untreated; ns = nonsignificant; *p* = *0.01–0.05, **0.001–0.01, ***0.0001–0.001, and ****0.00001–0.0001. (C) Immunoblot analysis of the soluble (Triton) or insoluble (SDS) fractions of HEK293T cells overexpressing P310Ltau^FL^-GFP after 48 h of treatment with samples of tau^4RD^ aggregated in buffer and in the presence of BCHE, cinnamaldehyde, or shikimic acid. The tau-5 antibody was used to assess tau protein. The first lane corresponds to cells without the treatment (NT). β-actin immunoblotting was used to verify equal protein loading in the Triton-soluble fraction.

Increasing evidence supports the hypothesis that tau pathology spreads through a prion-like mechanism. This process has been demonstrated in vitro and in cell culture models.^51^ Tau fibrils can be internalized by recipient cells, where they act as seeds by recruiting soluble tau and promoting templated fibrilization. To determine whether tau aggregates formed in the presence of BCHE, cinnamaldehyde, or shikimic acid could seed tau aggregation in cultured cells, we carried out a seeding-based aggregation assay using HEK293T cells transiently expressing the longest isoform of human tau with the P301L mutation, fused to a GFP tag at its C-terminus (Figure 5A). Aggregates of tau^4RD^, generated either with or without BCHE, cinnamaldehyde, or shikimic acid, were internalized into HEK293T cells using Lipofectamine. Two days post-treatment, the levels of soluble (Triton fraction) and insoluble (SDS fraction) tau were assessed by immunoblotting using the tau-5 antibody (Figure 5C). The levels of soluble tau were comparable across all samples, and tau^4RD^ fibrils formed in the absence or in the presence of shikimic acid-induced aggregation of tau in cells. In contrast, the cells treated with tau^4RD^ aggregates formed in the presence of BCHE or cinnamaldehyde showed a marked decrease in insoluble tau accumulation, evidenced by approximately 30% and 50% reductions in the blot band intensity, respectively (Figure 5C).

These data demonstrate that the mixture of molecules contained in BCHE and cinnamaldehyde can impair tau fibril formation, favoring off-pathway oligomeric species that exhibit reduced toxicity and seeding capacity in cell culture models.

### Concentrated Bioactive Molecules Perturb Tau-Polyanion Condensates and Impair Associated Aggregation

Like many intrinsically disordered proteins, tau can undergo liquid–liquid phase separation (LLPS) under specific conditions, forming dense-phase droplets (condensates) that coexist with a surrounding dilute phase.^52^ Emerging research indicates that tau engages in phase separation within cells and is present in membrane-less organelles like the nucleolus and stress granules.^16,53,54^ Tau phase separation has been observed in vitro under various experimental conditions.^17–19,55–57^ There is a general consensus that the condensed state can promote protein aggregation, providing an alternative to oligomeric intermediate–mediated deposition.^16,58,59^ Thus, targeting phase separation with phase modulators (including drugs, biomolecules, and nanomaterials) offers a novel therapeutic strategy for proteins involved in this process.^60–63^

Tau readily forms heterotypic condensates (complex coacervates) with polyanions, driven by multivalent electrostatic interactions. The highly sulfated polysaccharide heparin has a strong affinity for tau and can trigger both fibril formation and phase separation. Mixing tau^4RD^ with heparin resulted in the formation of micron-sized droplets, readily visible using bright-field microscopy (Figure 6A). To detect the structural conversion of tau^4RD^ from its condensed liquid state into β-sheet aggregates under LLPS conditions, we introduced ThT to mark the aggregates and examined the samples by fluorescence microscopy over a 24 h period (Figure 6B). After 1 h of incubation, the droplets disappeared, and no fluorescent assemblies were observed; however, fluorescent deposits were detected after 24 h, indicative of β-sheet aggregate formation. Analogous experiments were conducted in the presence of BCHE, cinnamaldehyde, and shikimic acid (Figure 6C–E). At low BCHE concentration (25 μg/mL), tau droplets remained visible in bright field after 1 h. Fluorescent deposits were detected after 24 h, similar in aspect to those observed in the absence of extract. A moderate BCHE concentration (50 μg/mL) had a limited effect on droplet appearance and aggregate formation. In contrast, high BCHE concentrations disrupted the formation of distinct spherical droplets, producing clustered structures after 20 min and larger visible assemblies after 1 h. Notably, these assemblies did not transform into ThT-positive deposits even after 24 h (Figure 6C). Cinnamaldehyde at 25 μg/mL showed stabilizing effects on droplets comparable to those observed with low BCHE concentration, whereas at 250 μg/mL, no ThT-positive aggregates were detected after 24 h (Figure 6D). The behavior of the condensates with shikimic acid was essentially unchanged compared with the control (Figure 6E).

**Figure 6:**
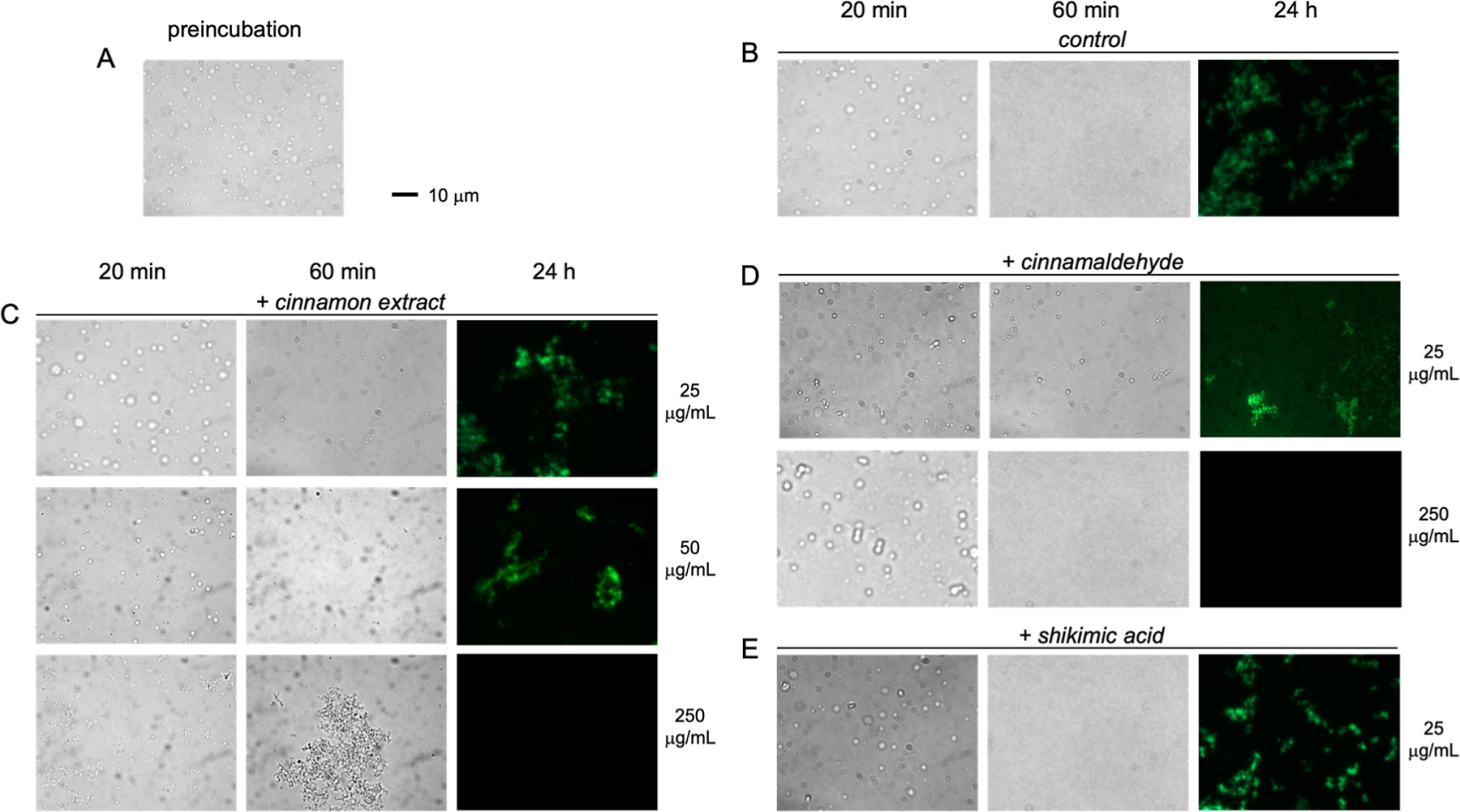
Liquid condensates of tau^4RD^/heparin. The formation and evolution of liquid droplets were monitored by microscopy. Representative fluorescence microscopy images are used to visualize fluorescent samples (marked with ThT), and bright-field images are displayed for samples that lack fluorescence. The tau^4RD^ protein was mixed with heparin, incubated for 5 min to equilibrate, and visualized (A) and then aliquoted and supplemented with ThT and (B) buffer as a control, (C) 25, 50, or 250 μg/mL BCHE, (D) 25 or 250 μg/mL cinnamaldehyde, or (E) 25 μg/mL shikimic acid. Images taken after 20 min, 60 min, and 24 h incubation are shown from left to right. The scale bar is 10 μm. Samples contained 50 μM*tau* ^4RD^, 11 μM heparin, 30 mM NaCl, and 1 mM DTT, dissolved in 20 mM sodium phosphate buffer at pH 6.0. Incubation and measurements were conducted at 37 and 25 °C, respectively.

It is reported that the RNA polyanion can induce tau condensation in vitro.^64^ Therefore, we aimed to investigate how bioactive molecules influence the phase separation of tau/RNA. The formation of tau^4RD^ condensates in the presence of polyU was examined using fluorescence microscopy (Figure 7A) after the introduction of a small quantity of Alexa488-labeled tau^4RD^. Spherical droplets observed after 5 min, with an average diameter of approximately 2 nm, evolved over time into a broad size distribution (Figure 7B–D). The fractional area covered by the droplets expanded at 40 min and contracted back by 60 min (Figure 7E). The introduction of a low concentration of BCHE (25 μg/mL) resulted in a decreased covered area relative to the control at 40 min; however, no significant differences were observed at 60 min (Figure 7C–E). In contrast, a high BCHE concentration (250 μg/mL) strongly perturbed LLPS, as the droplets appeared clustered together at both analyzed time points. In the presence of cinnamaldehyde, the droplets were only slightly perturbed relative to the control, whereas the addition of shikimic acid retarded condensate formation, which became observable at 60 min (Figure 7C–E).

**Figure 7:**
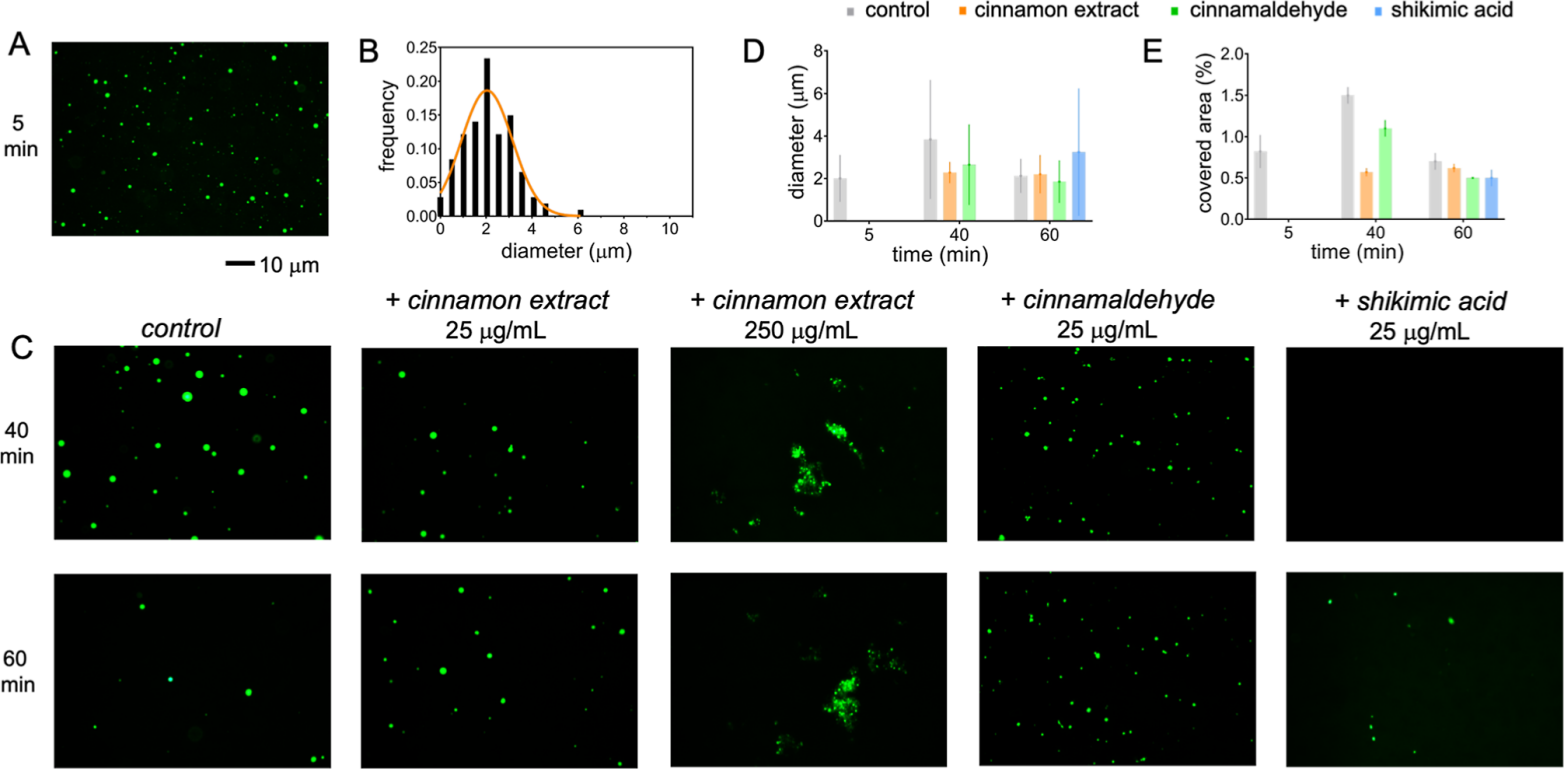
Liquid condensates of tau^4RD^/polyU. The formation and evolution of liquid droplets were monitored by fluorescence microscopy. Biomolecular components were mixed, incubated for 5 min to equilibrate (A), and then aliquoted and supplemented with (C) buffer as a control, 25 μg/mL BCHE, 250 μg/mL BCHE, 25 μg/mL cinnamaldehyde, or 25 μg/mL shikimic acid. Images taken after 40 and 60 min incubation are shown in the top and bottom rows, respectively. The scale bar is 10 μm. Samples contained 50 μM*tau* ^4RD^, 0.6 μM Alexa488-tau^4RD^, 125 μg/mL polyU, and 1 mM DTT, dissolved in 25 mM HEPES, pH 7.4. Incubation and measurements were conducted at 25 °C. (B) Size distribution of droplets corresponding to the sample as in panel (A); the Gaussian best-fit curve is shown in orange. (D) Droplet diameters and (E) % area covered by droplets, as determined from micrographs of samples as in panels (A) and (C) (in the presence of 25 μg/mL BCHE or isolated molecules); data are the mean ± SD (*n* = 20–100).

It emerges that BCHE and the tested bioactive molecules exert only modest effects on tau condensates at low to moderate concentrations. In contrast, higher concentrations produce pronounced perturbations at the level of condensates, as observed for BCHE, and prevent the formation of fibrillar aggregates under static conditions that promote LLPS, as seen for both BCHE and cinnamaldehyde. Overall, these findings indicate a clear concentration-dependent behavior. At sufficiently high levels, these molecules appear capable of interfering with the network of tau–polyanion interactions that underlies condensate formation and maturation. A mechanistic analysis is worth future work to uncover their potential in preventing aberrant aggregation driven by condensate formation.

In conclusion, our comprehensive study provides evidence that hydroalcoholic extracts from *C. cassia* buds, rich in cinnamaldehyde and shikimic acid, significantly modulate tau^4RD^ aggregation and phase separation, which are considered key pathological processes underlying Alzheimer’s disease and related tauopathies. Our in vitro analyses demonstrate that BCHE and its major component cinnamaldehyde effectively inhibit the formation and maturation of fibrillar tau aggregates, shifting the aggregation pathway toward nonfibrillar, off-pathway species. These alternative aggregates exhibit markedly reduced cytotoxicity in human neuroblastoma cells compared to canonical tau fibrils, as well as reduced seeding capacity, highlighting the potential of cinnamon-derived bioactive compounds as neuroprotective agents.

Furthermore, we observed that BCHE differentially affects tau’s LLPS behavior depending on the nature of cofactors involved (heparin versus RNA), underscoring the complexity of tau condensate modulation by small molecules. The capacity of BCHE at high concentration to impair fibril formation is also maintained in condensates, and the extract disrupts typical condensate morphology. Our results suggest a novel mechanism by which natural compounds may mitigate tau aggregation and its cellular consequences.

Moreover, NMR experiments revealed the binding of cinnamaldehyde to preformed tau fibrils, consistent with previous observations that cinnamate structural motifs mediate interactions with tau aggregates. These molecular insights are valuable for the future rational design of high-affinity tau fibril binders inspired by bioactive molecules in cinnamon.

Taken together, our findings highlight the potential of cinnamon-derived molecules as modulators of tau aggregation and phase behavior. Interestingly, the biomolecules found in BCHE have also been identified as ligands and inhibitors of Aβ aggregation and cytotoxicity,^32^ demonstrating multitarget properties that enhance their potential neuroprotective effects—especially valuable for tackling the complex nature of brain disorders. Given their favorable safety profiles, dietary availability, and ability to cross the blood–brain barrier, the natural compounds contained in cinnamon bud extract offer a great opportunity for the development of nutraceuticals to prevent or delay tau-driven neurodegeneration.

## Abbreviations

BCHE: *C. cassia* bud hydroalcoholic extract
CD: circular dichroism
GFP: green fluorescent protein
HEK293T: human embryonic kidney cells
LLPS: liquid–liquid phase separation
MWCO: molecular weight cutoff
NMR: nuclear magnetic resonance
polyU: polyuridylic acid
SD: standard deviation
STD: saturation transfer difference
TEM: transmission electron microscopy
ThT: thioflavin-T
UPLC-HRMS: ultraperformance liquid chromatography–high-resolution mass spectrometry
